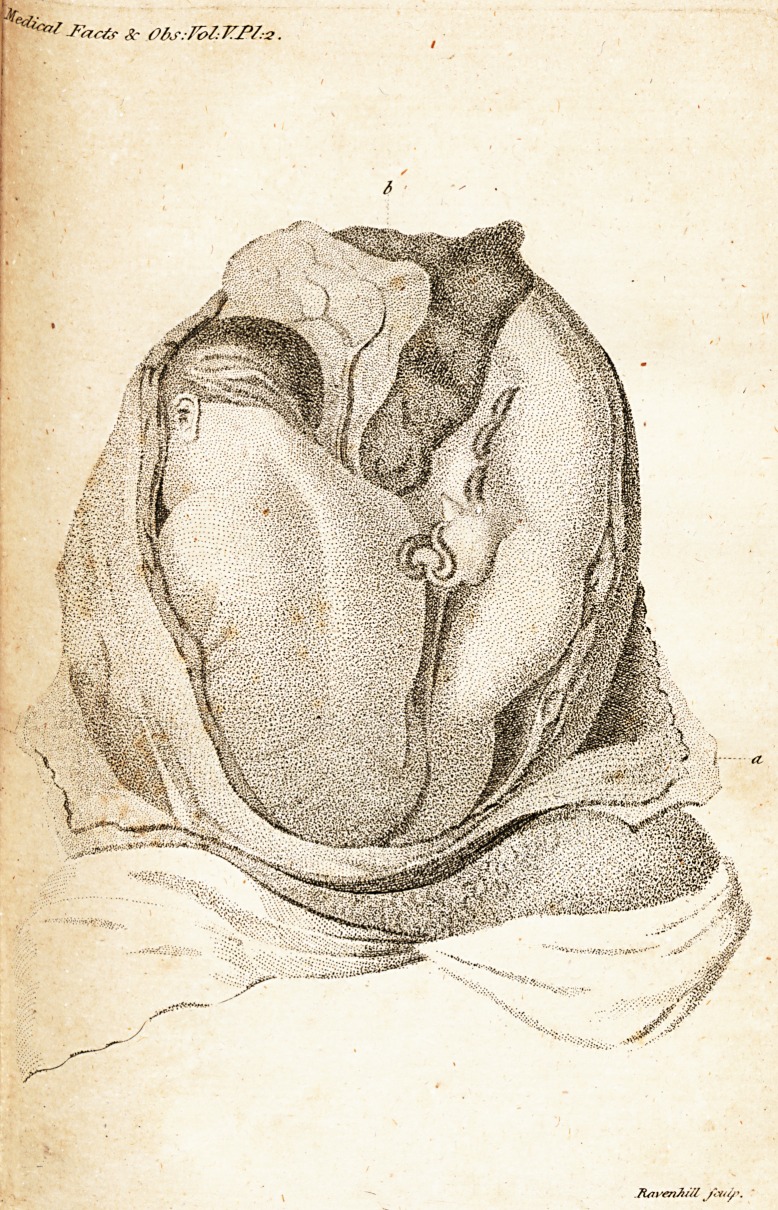# Case of Apoplexy in a Pregnant Woman; with Observations

**Published:** 1794

**Authors:** Philip Williams

**Affiliations:** Surgeon at Rugby in Warwickshire.


XI. *Caſe of Apoplexy in a pregnant Woman; with Obſervations. By Mr.* Philip Williams, *Surgeon at Rugby in Warwickſhire. Communicated in a Letter to* John Clarke, M. D. *Teacher of Midwifery in London; and by him to Dr.* Simmons.

THE following caſe, which I offer to you, appears to me to deſerve attention, not only from the remarkable ſituation of the children, but alſo from the circumſtances attending kidney much enlarged; and *f* to the termination of the left ureter.

Fig. II. ſhows the right kidney, uncommonly ſmall.

the the death of the patient. I ſhall firſt take notice of the latter, and then proceed to make ſome few remarks on the former.

A woman, about forty years of age, who was the mother of ſeveral children, had advanced to the laſt month of her pregnancy without any thing remarkable having occurred. One day, when ſhe was in apparent good health, and going about her uſual occupations, upon a ſlight exertion ſhe ſuddenly complained of a violent pain in her head, and had ſcarcely time to reach a chair, into which ſhe ſunk, and never ſtirred nor ſpoke afterwards.

On my coming to her, and finding her quite dead, I introduced my finger into the vagina, and found the os uteri dilated to the ſize of a crown piece; but was prevented, by the huſband's coming, (who would not ſuffer any thing to be done) from aſcertaining what part preſented.

Dr. Baillie and Mr. Cruikſhank afterwards examined, with me, the body at their diſſecting room. On opening the head coagulated blood was found in all the ventricles, and ſome had penetrated the very ſubſtance of the right optic nerve.

From the quantity of blood, (for there was between two and three ounces) and from the ſituation in which it was found, we need not wonder at the ſudden death of the patient. But it deſerves attention to enquire how far we ſhall be able to trace the cauſe of the extravaſation. The woman was by no means of a plethoric habit; neither was ſhe, at the time of her ſeizure, uſing any violent exertion.

Might not a diſpoſition to labour having come on, from the connexion known to exiſt between the brain and the uterus, produce a greater determination of blood to the brain than its veſſels were capable of bearing, and hence occaſion the rupture? That there had been an affection of the uterus, appears very probable from the ſtate in which the os uteri was found.

The preſentation has, I believe, never been before delineated. Both children, as will be ſeen by the plate *, preſent preternaturally; one with the breech, the other with the foot.

It may become a queſtion whether any difficulty would have occurred in the delivery? And alſo which of theſe children would have been born firſt had labour come on? It is moſt pro-

* See Plate II. in which the letters *a*, *a*, refer to the parietes of the abdomen, and *b* to the fundus uteri with the placenta adhering.

Ravenhill sculp.

bable that the woman would have been delivered without any thing unufual occurring or having been known of the fituation in which the children had lain in the womb. Had the labour been fuffered to proceed of its own accord, I think that the one whofe breech now prefents would have been born firft. For though the child, whofe foot prefents, (and which is under the breech of the other) has its head neareft to the fundus of the uterus, and confequently when the uterus came into action, the longitudinal fibres would have acted moft completely upon it; yet from the circular form in which it lies, its head, inftead of acting upon its own body, would probably have acted upon the head of the other child whofe breech prefents, and forcing it down, might either itfelf have gradually gone round, and, before the firft child had been born, making a complete evolution, have been born head firft; or it might have remained with its head where it now is, and after the birth of the other child have come with its feet firft.

One of the above circumftances, I think, would have happened had Nature been left to herfelf; but had the woman been attended in labour by one who was impatient of delay, it is moſt probable that when the foot was found preſenting, that child would have been brought firſt, in which caſe I do not think that any difficulty would have occurred in the delivery of the other.

*Query.* Might not the labour mentioned in the thirty-eighth chapter of Geneſis, v. 28, 29, and 30, have been ſimilar to the caſe which I have related; and that though the hand of one of the children was loweſt at the beginning of labour, yet as the moſt bulky part of the other child was below the body of this child, the hand receded, and the other child came firſt into the world?

## Figures and Tables

**Figure f1:**